# Grab Sampling or Passive Samplers? A Comparative Approach to Water Quality Monitoring

**DOI:** 10.3390/molecules31030529

**Published:** 2026-02-03

**Authors:** Caterina Cacciatori, Jackie Myers, Giulio Mariani, Bernd Manfred Gawlik, Vincent Pettigrove

**Affiliations:** 1 Aquatic Environmental Stress Research Group (AQUEST), RMIT University, Bundoora, VIC 3083, Australia; 2 Joint Research Centre (JRC), European Commission, 21027 Ispra, Italy

**Keywords:** passive samplers, Stir-Bar Sorptive Extraction (SBSE), pesticides, wide target screening, surface waters

## Abstract

Pesticide contamination poses significant threats to both humans and the environment, with residues frequently detected in surface waters worldwide. This study compares the effectiveness of passive samplers (POCIS and Chemcatcher) and grab sampling coupled with Stir-Bar Sorptive Extraction (SBSE) and Solid-Phase Extraction (SPE) for monitoring pesticides in surface waters. The comparative study was conducted at three sites in Victoria, Australia, representing different land uses. A total of 230 pesticides were screened, with 79 different pesticides detected overall. SBSE extracted the highest number of pesticides from grab samples, followed by SPE and passive samplers. The study highlights the complementarity of different sampling and extraction techniques in detecting a wide range of pesticides. The study also explores the suitability of these techniques for citizen science applications, emphasizing the importance of selecting appropriate methods based on specific research objectives and available resources. The findings underscore the need for a tiered approach, combining passive samplers for initial screening and grab sampling for quantitative analysis, to develop a robust monitoring strategy for protecting water quality.

## 1. Introduction

Pesticide contamination poses a significant threat to both humans and the environment [1]. Many pesticides are persistent, toxic and prone to bioaccumulation [2]. Their intensive and widespread application in agricultural and urban settings has led to substantial environmental and health concerns. Documented human health impacts range from acute intoxication to chronic diseases, including several cancers, Alzheimer’s disease, Parkinson’s disease, infertility, leukemia and diabetes [1]. Environmental impacts are equally concerning: pesticide runoff from agricultural and urban areas contributes to soil degradation, biodiversity loss, and contamination of aquifers [1].

Recent studies have consistently detected pesticides in surface waters worldwide. Extensive literature reviews have tried to understand the occurrence of pesticides in surface waters around the world [3,4]. According to the authors of [5], who scored pesticide occurrence and concentration globally, it appears that Africa, South America and Asia record higher numbers and concentrations of pesticides in surface waters than other regions. Europe, the US and China exhibit large regional variations. Among herbicides, atrazine, banned in Europe since 2007, is the most frequently occurring worldwide, followed by metolachlor. The most frequently detected insecticides are dimethoate and chlorpyrifos. Frequently detected fungicides include tebuconazole and carbendazim. Pesticides also frequently occur as mixtures, which are challenging to assess and may exert synergistic toxicity exceeding that of individual compounds [4]. The problem is exacerbated by the steady increase in pesticide use and sales over the last 50 years, which has boosted production since the 1990s [3,6]. In 1990, 1.2 kg/ha was applied compared to 1.8 kg/ha in 2019 [3]. Between 2019 and 2022, worldwide pesticide consumption was estimated to be between 2.7 and 4.1 million tonnes. In Australia, approximately 10,000 pesticide products were sold in 2024, with a total market value of AUD 4 billion [7]. Around 2000 active ingredients are currently authorized for use in the country [8], including compounds banned in many other countries, such as atrazine, simazine, terbacil, and metolachlor [8,9]. Despite this large number, only a small subset is routinely monitored in water. For instance, the Australian Government’s National Measurement Institute (NMI) screens for approximately 230 pesticides [10]. The list of screened compounds can be consistently smaller in other counties, a common worldwide limitation on the acquisition of water quality data collection. Several studies from Europe report that surface water monitoring for pesticides does not capture seasonal variations nor cover for smaller water bodies, often missing more localized areas which might be under contamination stressors [11,12,13].

The escalating use of pesticides and increasing human and ecosystem exposure underscore the need for more effective monitoring of pesticides in surface waters. Wide-screening methods and the incorporation of alternative data sources can enhance pesticide prioritization, enabling monitoring schemes that are more region-specific, feasible and fit for purpose. The choice of monitoring techniques depends largely on the characteristics of the target compounds, source water properties [2], and the availability of human, laboratory and financial resources [14]. 

Passive sampling approaches offer a cost-effective, long-term monitoring option. Devices such as the Polar Organic Chemical Integrative Sampler (POCIS) and Chemcatcher accumulate contaminants on different absorbing materials, such as membranes and bulk sorbents, over extended deployment periods, usually ranging from 10 to 30 days [15]. These methods have been successfully applied in south-eastern Australia. For example, ref. [16] detected 21 pesticides across 22 waterways, with the greatest number of pesticides occurring in streams receiving runoff from intensive agriculture. Similarly, ref. [17] reported up to 70 pesticides in more than 100 urban wetlands in Melbourne, Australia. Passive samplers are lightweight, require only two site visits (deployment and retrieval) and provide a more representative picture of fluctuating contamination compared to traditional grab sampling [17,18]. However, challenges include potential loss or vandalism of deployed devices as well as site-specific deployment considerations [2]. While passive samplers integrate larger volumes of water, can capture ultra-trace-level contaminants and transient contamination events, calibration remains complex, and results cannot be compared to environmental quality standard guidelines or regulations [17]. Ref. [19] reported poor agreement between POCIS and grab sample concentrations of Endocrine-Disrupting Chemicals (EDCs), limiting the acceptance of passive samplers as standalone monitoring tools under the European Union Water Framework Directive [2]. In contrast, grab sampling provides quantitative concentrations aligned with regulatory guidelines, but it is limited to small discrete water volumes that may not capture temporal variability and might miss short-time peak concentrations. This can be problematic for pesticides, whose occurrence and transport are influenced by application times, weather and runoff events [20]. Furthermore, pre-concentration and extraction procedures, such as Solid-Phase Extraction (SPE), often require large volumes of solvents or samples [14,21]. Newer micro-extraction methods, such as Stir-Bar Sorptive Extraction (SBSE)—a widely implemented sorbent-based micro-extraction technique [14]—address some of these limitations by reducing solvent requirements and simplifying workflows, while maintaining high analytical sensitivity [22]. SBSE is based on the use of a stir bar, a small glass magnet (10 or 20 mm), coated with an absorbent layer (0.5 or 1 mm) of polydimethylsiloxane (PDMS) or polyethylene glycol (PEG), on which compounds with specific properties are absorbed by magnetic stirring [14]. SBSE streamlines sample processing, reducing procedural errors and analyte losses often associated with SPE [14]. SBSE sensitivity also results in lower LODs compared with traditional techniques [20]. On the other hand, SPE offers versatility in terms of a wide polarity range of compounds extracted, which is limited in SBSE by the type of absorbent phase. The expected recovery of both extraction techniques can be compromised by matrix effects [23].

Alternative data sources are also gaining attention. Citizen science is increasingly recognized by international and national organizations as a more cost-effective and socially beneficial approach to water quality monitoring [11,12,24,25,26]. Citizen science is deemed relevant in water quality monitoring because it can support the generation of valuable data at a higher spatial and temporal resolution than traditional monitoring [12]. Furthermore, there are many positive impacts of citizen science on society. Citizens’ involvement raises environmental awareness, connects communities and policymakers, and fosters well-being [27,28,29]. Most existing citizen science for water quality programs focuses on simple on-site measurements, such as pH, turbidity, and nutrient measurements [30,31,32]. To date, only two initiatives—Pesticide Detectives by the RMIT Aquatic Environmental Stress Research Group (AQUEST) [33] and Pesticide Watch by Deakin University [34]—have tried to integrate pesticide screening with citizen science. This highlights the opportunity to further evaluate the potential of applying advanced monitoring methods, such as passive samplers and micro-extraction techniques, in citizen science contexts. 

Although several studies have examined the complementarity of passive samplers and their comparison with grab sampling coupled with SPE [15,17,35,36], far fewer have evaluated passive samplers against SBSE for pesticide monitoring in surface waters [37]. Moreover, no study has systematically compared these methods across dimensions such as sampling time, compound selectivity, sample preparation and processing requirements, material use, and logistical practicality, especially in citizen science applications. Therefore, this study aims to (1) evaluate the effectiveness of different sampling (three passive samplers and grab sampling) and extraction techniques (SBSE and SPE), (2) determine advantages and disadvantages of grab and passive samplers, and (3) assess the practicality, accessibility, and methodological suitability of these techniques for potential application in citizen science. To our knowledge, this is the first study to systematically evaluate the trade-offs between passive sampling and grab samples coupled with micro-extraction (SBSE) within the specific operational constraints of a citizen science monitoring initiative, thereby bridging the gap between rigorous analytical chemistry and participatory science. The baseline comparison described in this paper provides insightful assessments of different techniques for “The Gems of Water” initiative [38], a joint initiative between RMIT AQUEST in Australia and the European Commission’s Joint Research Centre (JRC), designed to deepen the understanding of pesticide occurrence in water through citizen science.

## 2. Results and Discussion

### 2.1. Performance of the Different Sampling and Extraction Methods in Terms of Pesticides Detected

**Figure 1:**
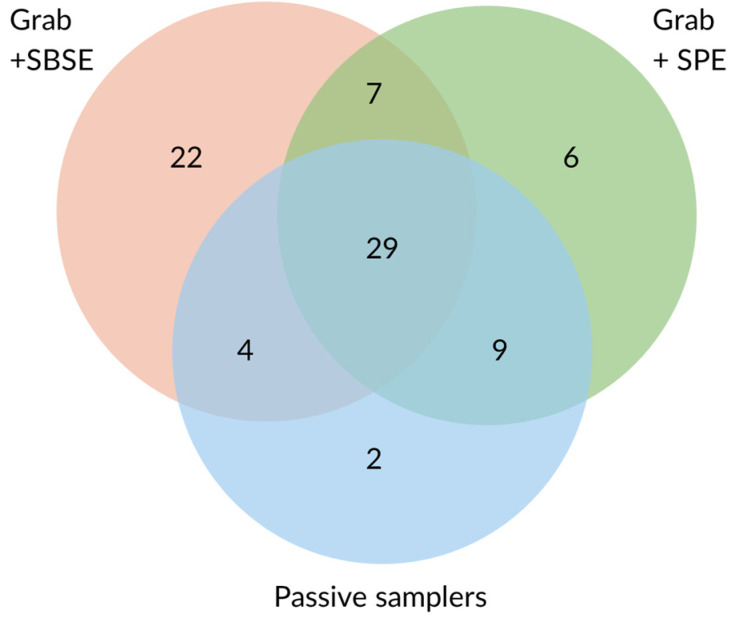
Number of pesticides detected by grab sampling (SPE and SBSE) and passive samplers.

A total of 79 different pesticides were detected overall. Most compounds were extracted from grab samples by SBSE (62), followed by SPE (51), and by passive samplers (44) (Figure 1). More on the comparison between SPE- and SBSE-extracted samples can be found in [39]. POCIS detected the highest number of pesticides amongst the passive samplers (40), followed by Chemcatcher with SDB disks (37). The lowest number of pesticides was detected with Chemcatcher with HLB disks (31). Only around half of the pesticides detected by SBSE and SPE were picked up by the passive samplers. Among the pesticides detected in all SBSE- and SPE-extracted samples, 54.4% and 87.5%, respectively, were detected by all passive samplers. The highest percentage of concordance between grab-sample SPE and passive samplers might be linked to the common use of HLB as a sorbent material. Of the compounds found in less than 60% of SBSE- and SPE-extracted weekly samples, only 24.1% and 30% were detected by all passive samplers, respectively. The higher frequency of pesticides extracted in grab samples somewhat corresponded to a higher number of compounds being detected in parallel by passive samplers, probably due to the higher concentrations at which those compounds occur or due to more consistent pollution over the weeks. 

Among the compounds not detected by the passive samplers, the majority were detected at trace levels (0.1–8.5 ng/L). About 10 of those pesticides had a Log K_ow_ > 5. Conversely, 11 compounds extracted by the passive samplers were not detected by SBSE. These compounds are relatively polar with an average Log K_ow_ of 2.9. Only two compounds extracted by passive samplers were not extracted by SPE. SPE is performed with HLB cartridges, thus employing the same sorbent medium as Chemcatcher with HLB disks and POCIS. Most compounds detected by passive samplers were commonly detected among the three samplers. 

According to Table 1, the median Log K_ow_ and Log K_ow_ 1st quartile values are similar for the compounds detected by passive samplers, while SBSE and SPE are associated with slightly higher median and quartile values. In total, 75% of the compounds detected by SBSE have a log K_ow_ above 5.3.

Ref. [35] reported a higher median Log K_ow_ for pesticides detected by POCIS (4.6) than for those detected by Chemcatcher with SDB disks (3.2). No such difference was reported in our study, where the median Log K_ow_ of the detected compounds was 3.7 for both techniques. The POCIS ideal polarity range is reported to be between 0 and 4 [40], while Chemcatcher SDB disks have been reported to extract compounds in the hydrophobicity range between 2 and 6 [41]. Chemcatcher HLB disks use the same absorbent material as POCIS and might therefore be more efficient in the extraction of semi-polar to polar compounds, while overcoming the possible POCIS solvent loss due to its bulky nature, which reduces the active sampling area [16]. The pesticide screen used in this study, which included compounds with median Log K_ow_ values of 4.0 and where 75% of compounds had a log K_ow_ > 3.4, could have played a role in the lower numbers of pesticides detected by passive samplers compared to grab samplers. Ultimately, various studies have confirmed the complementarity of POCIS and Chemcatcher in terms of types of pesticides detected [16,17,35], supporting the use of multiple passive samplers to increase the screening capacity. These first observations hint at a complementarity among SBSE- and SPE-extracted grab samplers and passive samplers. SBSE appears to extract more compounds at trace levels, while SPE and passive samplers extract compounds with a wider polarity, thanks to their common absorbent material (HLB).

### 2.2. Performance at the Different Sampling Sites

**Figure 2:**
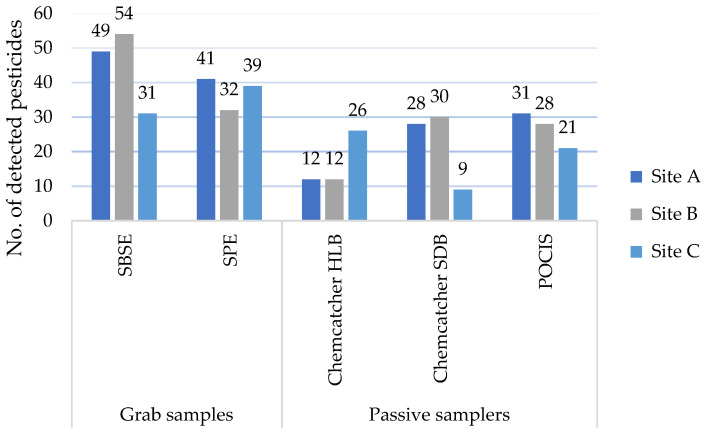
Summary of results of pesticides detected at sample sites A, B, and C, deploying Chemcatcher with Styrene–Divenylbenzene (SDB) and Hydrophilic–Lipophilic Balance (HLB) disks, a Polar Organic Chemical Integrative Sampler (POCIS), and grab sampling performed by Stir-Bar Sorptive Extraction (SBSE) and Solid-Phase Extraction (SPE).

Regarding the performance of the different sampler types at the sites (Figure 2), the complete list of compounds found at each site above the LOQ with indications of the frequencies for multiple grab-sampling events can be consulted in Table S5 of the Supplementary Materials. At Sites A and B, grab samples extracted the highest number of pesticides (SBSE (49; 54) and SPE (41; 32)). The passive sampler counts were similar at the two sites for POCIS and Chemcatcher with SDB disks (28–31). Chemcatcher with HLB disks detected around 10 fewer pesticides (12) than the other passive samplers at Sites A and B. Unlike the other sites, Site C had a more even distribution of pesticides extracted by grab and passive samplers. In the grab samples, 31 and 39 pesticides were extracted respectively with SBSE and SPE. Notably, Chemcatcher using HLB disks performed best at Site C, detecting up to 25 pesticides, in comparison to Chemcatcher with SDB disks (9) and POCIS (21). This might be linked to the matrix condition at the site or to desorption mechanisms, as have been observed for SBSE [39]. Indeed, Site C reported a turbidity of 11.6 NTU as compared to Site B’s level of 6.1 NTU and Site A’s of 2.1 NTU. SBSE detected the highest number of pesticides at all sites, except at Site C. The lower performance of SBSE at Site C has been discussed in [39] and has been linked to potential desorption and matrix effects, linked to turbidity, at the different sample sites.

Due to limited observations, it is hard to understand how Chemcatcher with HLB disks detected more compounds than the other two passive samplers at Site C. While this cannot be directly linked to land use, it shows that performance might be affected by different elements and thus combined use can help to gain a better overview. Eventually, beyond Site C, Chemcatcher with HLB disks appears to be the least performant among the passive samplers, both in terms of the number of detected compounds as well as in terms of complementarity with other samplers, as no compound was solely detected by this sampler type. Beyond the selectivity of absorption and extraction methods, pesticide load and matrix effects might play a role in the performance of the samplers.

### 2.3. Advantages and Disadvantages of Passive Samplers and Grab Samples

Previous sections highlight the complementarity of grab sampling and passive samplers in terms of the type of information that can be obtained. Passive samplers provide qualitative information over a longer time span, while grab samples can indicate numbers and concentrations of pesticides found in a set volume for an instantaneous sample. In this study, the recovery of pesticides by passive samplers reflected the trend of pesticides detected in grab sampling, where a more consistent presence over the weeks for grab sampling was related to a higher probability of detections in passive samplers. From a logistical perspective, passive samplers present some advantages. Even if deployed over a four-week period, they limit the visits to the site to only two, for deployment and retrieval. At the same time, these steps might require entering the water course, which can be risky. Visits to the sites can quickly increase when collecting grab samples, making logistics more complex. On the other hand, frequent site visits provide a chance to check the water body over the sampling period, which could be beneficial to assess unexpected loss of passive samplers, a change in water level or unwanted human removal.

Expanding the comparison to the preparation and extraction steps required for the different techniques deployed in this study can help assess the appropriateness of each depending on context and for citizen science initiatives. Ref. [2] suggests a series of criteria which can be considered when choosing a sampling and extraction method. These include sample preparation times, solvent use, selectivity, precision and sensitivity. Based on the presented study, Table 2 reports a basic comparison of the techniques used, with reference to materials, sample preparation and processing, sampling, and analysis.

The samplers and sampling preparation and processing steps are more labor-intensive for passive samplers; they require more material, solvent use and time (Table 2). Stir bars might also seem to require longer processing steps, but they are mostly passive, where active steps are reduced to move the stir bar in the conditioning unit and to add it to the water sample for extraction. Contamination risk and sample loss are higher for POCIS and SPE, due to the bulk character of POCIS sorbents and due to additional steps required, such as extraction with HLB cartridges and pre-filtering of the grab samples. In the case of SBSE, contamination might come from the passive nature of the PDMS rubber, which can potentially absorb contaminants found in the surrounding environment.

Passive sampler devices are relatively cheap, can screen for a wide polarity range and allow for overtime monitoring. Disadvantages are linked to the difficulties of assembly and use of solvents, which require specialized personnel. Additionally, Chemcatcher absorbed disks need to remain wet before and after deployment. Furthermore, POCIS has been associated with sorbent loss during sample preparation and implementation, due to its bulk form. To overcome POCIS limitations, ref. [36] has proposed the application of HLB disks in Chemcatcher, which use the same sorbent material but fixed in a glass fiber filter. HLB disks were deployed in Chemcatcher in this study as well. While they solve the sorbent loss issue of POCIS, the thickness of the HLB disks makes the assembly even more difficult. Solid-Phase Extraction (SPE) offers a higher extraction efficiency and lower solvent usage, but it is expensive and the procedure is long and complex and labor-intensive [14]. Just like passive samplers, SPE requires specialized personnel to perform sample preparation and handling protocols. SBSE’s main advantages are linked to the ease of application and the reduced use of solvents. Nonetheless, SBSE has limitations, including matrix effects and limited applicability to hydrophobic or polar contaminants [23]. It is a rather costly technique, as it requires specific equipment for conditioning of the stir bars and for automatic stir-bar desorption. Finally, it is a destructive technique, with the whole sample being desorbed at once. Ref. [39] also found that SBSE tends to underestimate concentrations compared to SPE. Further study relating to how matrix effects impact stir-bar absorption is suggested, as well as investigation of how to possibly expand the technique to wider polarity ranges.

### 2.4. Suitability for Applications in Citizen Science

All the techniques examined have been demonstrated to be suitable for citizen science applications to monitor pesticides in surface waters. The selection of an appropriate method should be guided by the specific research objectives and available financial and human resources. Grab sampling coupled to SBSE is particularly suitable for citizen science due to its simplicity, safety and portability, and it has already been deployed in various citizen science exercises around the world [42,43]. A key advantage is its ability to foster citizen engagement, allowing participation in both the sampling and extraction phases. However, it is a relatively costly technique that requires specialized laboratory equipment for conditioning, desorption and analysis, which may not be universally available. Grab sampling with SPE represents a mid-range option. It is less expensive than SBSE and provides robust quantitative data. A limitation for citizen engagement is that participation is typically restricted to sample collection, with extraction and analysis performed in a laboratory. Passive samplers (Chemcatcher and POCIS) are ideal for enhancing data collection over extended temporal scales within a watershed. They are highly cost-effective and require relatively inexpensive, common laboratory equipment. Citizen participation is generally limited to deployment and retrieval. While these steps can represent logistical challenges, they can be easily overcome by providing clear instructions and training and sampling procedures. The fundamental difference in data output must also be considered in the choice. Passive samplers provide qualitative information over their deployment period, whereas grab sampling delivers a quantitative snapshot of contaminant concentrations at a specific moment. A combined or tiered approach is ideal, where the complementarity of the methods allows identification of a wider range of pesticides. For instance, passive samplers can be used for an initial wide screening to identify contamination hotspots, followed by grab sampling for quantitative analysis at specific times and locations. This provides a more comprehensive understanding of local contamination, while engaging citizens with a variety of scientific techniques. 

## 3. Materials and Methods

### 3.1. Sampling Sites

**Figure 3:**
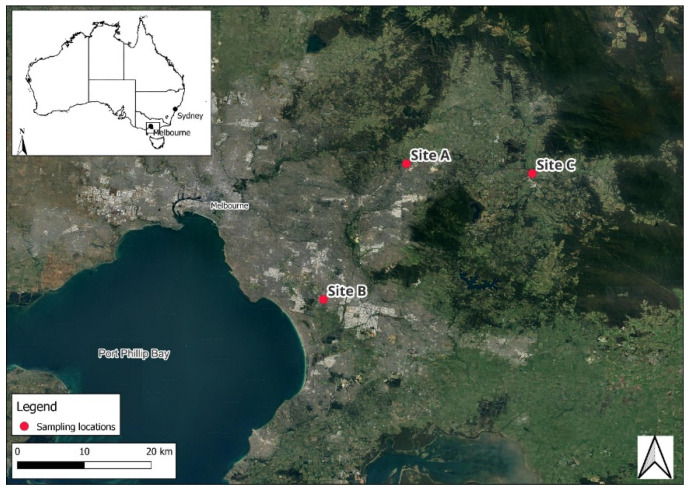
Map of sampling locations in eastern Melbourne, Victoria, Australia.

This study was conducted at three sampling sites in Victoria, south-eastern Australia. The sites, referred to as Site A, Site B, and Site C (Figure 3), were selected to represent distinct land uses, including a residential area downstream of a wastewater treatment plant outlet (Site A), an industrial urban residential neighbourhood (Site B), and a stream adjacent to horticultural and pastoral lands (Site C). Sample site coordinates can be consulted in Supplementary Table S1.

**Figure 4:**
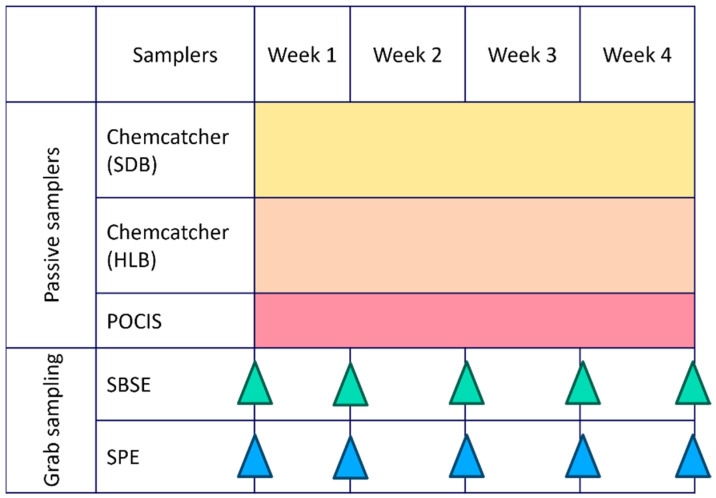
Deployment scheme of passive samplers (Chemcatcher with Styrene–Divenylbenzene (SDB) and Hydrophilic–Lipophilic Balance (HLB) disks and Polar Organic Chemical Integrative Sampler (POCIS)) and grab sampling performed by Stir-Bar Sorptive Extraction (SBSE) and Solid-Phase Extraction (SPE) monitoring over 4 weeks.

A combination of passive samplers and grab sampling paired with two different extraction methods was evaluated. Grab water samples (1 L and 100 mL) were collected in duplicate on a weekly basis over a four-week period during the summer season (8 February 2023–8 March 2023), resulting in five sampling events at each site. Blank samples, consisting of spiked Milli-Q water, were processed weekly during extraction to ensure quality control. During the same period, three different passive samplers were deployed in duplicate at each site for four weeks (Figure 4). Field and laboratory blanks were included to ensure quality control during the preparation, deployment, and retrieval of the samplers. In addition, in situ water quality parameters (pH, turbidity, electro-conductivity, dissolved oxygen, and temperature) were measured at each sampling event.

### 3.2. Chemicals

A total of 230 pesticides were screened, representing three major classes: insecticides (41.7%), fungicides (24.3%), and herbicides (19.6%) (Table S2, Supplementary Materials). A mix of solutions of all pesticides screened for were used in the method development. The solutions purchased are part of the GC Multiresidue Pesticide Standard mixes produced by the Restek Corporation (Bellefonte, PA, USA). Out of the 230 pesticides screened, a standard solution was not available for 12 compounds, namely, 2,4,6-tribromoanisole, 2-chloronaphthalene, 2-phenylphenol, 4,4′-methylenebis(N,N-dimethylaniline), acetophenone, dichlorbenzamide, chlorocre-sol, epoxiconazole, isoxadifen, phthalimide, quinoline, and resorcinol. They were excluded from quantitative analysis (indicated in italics in Table S2, Supplementary Materials). The internal standard solution contained *trans*-Nonachlor (^13^C_10_, 98%; Cambridge Isotope Laboratories Inc., Tewksbury, MA, USA) Further details on the solutions used are given in [39]. Pesticide residue analysis-grade methanol, ethyl acetate and toluene were used as solvents (Merck KGaA, Darmstadt, Germany).

Passive samplers included POCIS and Chemcatcher. POCIS used an Oasis HLB bulk sorbent (200 mg, 30 µm; Waters, Amberley, Ipswich, QLD, Australia) enclosed between two polyether sulfone (PES) membranes (0.1 µm, 90 mm; Sterlitech, Auburn, WA, USA). Chemcatchers were equipped with either Empore SDB-XC disks (47 mm; Phenomenex, Lane Cove, NSW, Australia) or HLB disks (Atlantic HLB 47 mm; Horizon Technology, Lake Forest, CA, USA), both covered by a PES diffusion-limiting membrane (47 mm; Sartorius Stedim Biotech GmbH, Göttingen, Germany).

For SBSE, stir bars coated with polydimethylsiloxane (PDMS; 2 mm × 0.5 mm) were obtained from Gerstel (Mülheim an der Ruhr, Germany). Water samples for SPE were first filtered using GF/C filters (47 mm, Whatman, Florham Park, NJ, USA) and then extracted with Oasis HLB cartridges (Waters Corporation, Milford, MA, USA).

### 3.3. Sampler Preparation

Stir bars were conditioned for 5 h at 330 °C before use. No preparation is required for SPE-extracted grab samples before extraction. The preparation of POCIS and SDB-XC Chemcatcher samplers followed the protocol in [17]. In the POCIS samplers, 0.2 g powder HLB sorbent was sandwiched between two PES membranes in a metal casing. Polyethersulfone membranes were activated by soaking in methanol (30 min) followed by Milli-Q water (30 min). No treatment was applied on the HLB solvent.

Chemcatcher Empore SDB-XC disks were soaked in methanol (30 min), followed by Milli-Q water (30 min), for activation. Each Chemcatcher housing contained an SDB-XC disk covered by a PES membrane. To prevent drying of the disks, they were moistened with Milli-Q water and capped. 

The HLB disks were conditioned in high-density polyethylene membrane (HDPE) holders by rinsing them sequentially with ethyl acetate (2 × 30 mL), followed by methanol (2 × 30 mL) and Milli-Q water (1 × 20 mL), according to the protocol modified from [36,44]. These methods were tailored to the target compounds and the instrumentation available, namely, Gas Chromatography Quadruple Time-of-Flight High-Resolution Mass Spectrometry (GC-QToF-HRMS). After conditioning, the disks were dried under vacuum and placed in the Chemcatcher casing and covered with a PES membrane. Owing to the greater thickness of the HLB disks, the casings were additionally secured with waterproofed tape to ensure closure. For quality assurance, one laboratory blank was kept open during the preparation of each type of passive sampler. A comprehensive graphical abstract depicting the sample preparation and processing can be found in Supplementary Material Table S3.

### 3.4. Sample Processing

The graphical abstract shows how the samples were extracted. The extraction of water samples was performed using two primary methods: SBSE and SPE. For SBSE extraction, a 100 mL water sample is stirred with a magnetic stir bar, on which rubber-coating contaminants absorb. The total extraction procedure lasts 5 h. For SPE extraction, a 1 L sample is first filtered and then loaded on an HLB cartridge, a process that takes approximately one hour per sample. Extraction of grab samples by SBSE and SPE has been described in detail in [39]. For the passive samplers, processing followed the established literature methods. The POCIS and SDB Chemcatcher were processed according to [17], while the HLB Chemcatcher samples followed the protocol in [44]. Before elution, Chemcatcher SDB and HLB disks were dried on a hot plate at 35 °C for 1 to 1.5 h. SDB-XC disks were then eluted using ethyl acetate (2 × 10 mL), whereas HLB disks were eluted using an automatic extractor system (PrepLinc AS4, J2 Scientific, Columbia, MI, USA) using ethyl acetate (3 × 20 mL). Similarly, POCIS HLB sorbent was transferred into empty SPE cartridges under vacuum and eluted according to the protocol described in [39]. Prior to extraction (grab-sample SBSE and SPE) and prior to elution (POCIS and Chemcatchers), the internal standard (^13^C_10_ *trans*-Nonachlor), diluted in acetone (1 mL), was added to achieve a final concentration of 5 ng/L for quality control and recovery calculation. The passive-sampler and grab-sample SPE eluate was concentrated by centrifugation (SPEED, approx. 30 min per sample) and reduced under a nitrogen flow to reach a final volume of 100 µL.

### 3.5. Chemical Analysis

All samples were analyzed using a Gas Chromatograph 8890 coupled with a Quadruple Time-Of-Flight 7250 mass spectrometer (Agilent Technology, Santa Clara, CA, USA). SBSE samples were automatically transferred by a Multi-Purpose Sampler (MPS, Gerstel, Germany) for desorption in a Thermal Desorption Unit (TDU2, Gerstel, Germany), while all other extracts were injected directly. Separation was performed on an HP-5MS UI capillary column (length = 30 m, internal diameter = 0.25 mm, film thickness = 0.25 µm; Agilent Technology, CA, USA). Injection and desorption parameters are detailed in the Supplementary Materials (Table S4).

Quantitative methods, including retention times, quantifier and qualifier ion masses for each compound, and the internal standard (^13^C_10_ *trans*-Nonachlor), are provided in Supplementary Table S2. Details on grab-sample quantification, calibration curve generation and determination of Method Detection Limits, Limits of Detection and Limits of Quantification are the same as those reported in [39]. Data acquisition and analysis were performed using Quantitative Agilent Software (version 12.1).

Data analysis was performed in Microsoft Excel (Version 2502) and Spyder (Python 2.7).

## 4. Conclusions

The findings of this study highlight several key insights into the effectiveness and practicality of various sampling and extraction techniques for monitoring pesticides in surface waters in the context of a citizen science activity. First, Chemcatcher HLB disks were excluded from further application due to the difficulties encountered during their assemblage and their limited performance in the detection of pesticides and complementarity with other passive samplers. Except for Chemcatcher HLB disks, the comparison of different techniques, including passive samplers and grab sampling, demonstrated that each method detects different sets of compounds, emphasizing their complementarity in monitoring applications. This complementarity is crucial for a comprehensive understanding of pesticide contamination. While passive samplers do not provide quantitative concentrations, they are invaluable for identifying compounds of concern over a longer deployment time and supporting prioritization for follow-up quantitative analysis. However, passive samplers might miss compounds present at trace levels, which is an important consideration when designing a monitoring strategy. A tiered approach may be appropriate, where passive samplers are employed during an initial screening stage, followed by grab sampling with a stir bar or Solid-Phase Extraction to generate quantitative information on a list of prioritized compounds. Additionally, land use should be considered when deciding a monitoring strategy. In catchments dominated by continuous point-source contamination, such as the Site B wetland or sites located near water treatment plant outlets like Site A, less frequent grab sampling may be sufficient. In contrast, catchments affected by variable or diffuse sources, such as agricultural areas like Site C, may require sampling at shorter intervals to capture episodic contamination events. Finally, for citizen science applications, the choice of one or another sampling and extraction technique should be based on the purpose of the initiative. If the aim is to bring citizens closer to science through increased engagement in the analytical process, grab SBSE might be more appropriate. If the focus is rather on complementing data on water quality, limited sampling and passive samplers might be satisfactory. The financial resources available might also help determine which technique is fit for purpose, with passive samplers being a rather cheap technology while grab-sampling SBSE requiring expensive and specific laboratory equipment for analysis. Overall, this study underscores the importance of selecting appropriate sampling and extraction techniques based on the specific characteristics of the monitoring site, the types of contaminants of interest, and citizen engagement activity. By leveraging the strengths of different methods, a more robust and comprehensive monitoring strategy can be developed to understand ongoing surface water contamination by pesticides. In the future, in terms of methodology, such a project would benefit from expanding the list of screened parameters beyond hydrophobic pesticides to wider polarity ranges, especially considering stir-bar sorbent PDMS phases. Additionally, enhancing the method to detect a broader array of metabolites and transformation products would provide a more comprehensive analysis of the samples. In terms of citizen science and policy applications, future work could explore establishing a tiered joint approach; this would involve citizen science groups in the preliminary screening stage, followed by local water quality monitoring authorities for subsequent in-depth contamination analysis.

## Figures and Tables

**Table 1:** Simple statistics for Log K_ow_ of detected pesticides by sample type.

	Sample Type				
	Passive Samplers			Grab Samples	
Log K_ow_	Chemcatcher HLB Disk	Chemcatcher SBD Disk	POCIS HLB Powder	SBSE	SPE
Median	3.7	3.7	3.7	4.1	3.8
Average	3.7	3.6	3.7	4.3	3.8
1st Quartile	2.9	2.8	2.9	3.4	3.0
3rd Quartile	4.5	4.2	4.4	5.3	4.3

**Table 2:** Summary of comparison of samplers and extraction techniques regarding material used, sample preparation and processing, and analysis. Solvent volumes and times were estimated based on this study and therefore calculated for ca. 12 samples. Abbreviations are reported at the end of the table.

**Material**	Sampler type	Chemcatcher	Chemcatcher	POCIS	SBSE	SPE
	Absorbent material	HLB disk	SDB disk	HLB bulk	PDMS	HLB cartridge
	Selectivity (Log K_ow_)	−5–8	2–6	0–4	3–7	−5–8
**Sampler &** **sampling preparation**	Steps	(1) Cleaning of casing (2) Cleaning of PES membrane (3) Pre-cleaning and activation of disks	(1) Cleaning of casing (2) Cleaning of PES membrane (3) Pre-cleaning and activation of disks	(1) Cleaning of casing (2) Cleaning of PES membrane	(1) Cleaning sampling bottles	(2) Cleaning sampling bottles
	Estimated duration	1 h	45 min	45 min	15 min	15 min
	Solvent use (est. volume)	Methanol (1 L); MilliQ-water (350 mL); ethyl acetate (60 mL)	Methanol (2 L); MilliQ-water (500 mL)	Methanol (1 L); MilliQ-water (300 mL)	Aceton (ca. 100)	Aceton (ca. 200 mL)
**Sampling**	Exposure time	4 weeks	4 weeks	4 weeks	Instantaneous	Instantaneous
	Sample volume	Unknown	Unknown	Unknown	100 mL	1 L
	Sampling casing	1 PES membrane PTFE casing	1 PES membrane PTFE casing	2 PES membranes Stainless steel casing	None	None
	Control over monitoring	Low	Low	Low	High	High
	Practicality	Medium	Medium	Medium	Medium	Medium
**Sample processing**	Steps	(1) Drying of disks (2) Elution (3) Extract concentration	(1) Drying of disks (2) Elution (3) Extract concentration	(1) Extraction with HLB cartridges (2) Elution (3) Extract concentration	(1) Thermal conditioning (2) Extraction by magnetic agitation	(1) Filtering of samples (2) Extraction with HLB cartridges (3) Elution (4) Extract concentration
	Estimated duration	8 h	8 h	8 h	10 h	8 h
	Solvent use (est. volume)	Ethyl acetate (60 mL) Acetone (1 mL)	Ethyl acetate (20 mL) Acetone (1 mL)	Ethyl acetate (10 mL) Acetone (1 mL)	Acetone (1 mL)	Ethyl acetate (10 mL) Acetone (1 mL)
	Contamination risk	Low	Low	Medium	Medium	Medium
**Analysis & results**	Injected volume of sample volume	N.A.	N.A.	N.A.	100 mL	20 mL
	Type of information	Long-term chemical exposure	Long-term chemical exposure	Long-term chemical exposure	Instantaneous, concentration	Instantaneous, concentration
	Units	ng/tot	ng/tot	ng/tot	ng/L	ng/L
**Citizen science**	Suitability	Limited (deployment and retrieval)			High (sampling and extraction, portable, laboratory-safe)	Limited (sampling)
	Advantages	Wide polarity range Cheap Continuous monitoring	Wide polarity range Cheap Continuous monitoring	Wide polarity range Cheap Continuous monitoring	Quantitative Easy to apply	Wide polarity range Quantitative Widely used standardized method
	Disadvantages	Difficult to assemble PES membrane needs to be humid High solvent use Requires manual skills Qualitative Specialized personnel for laboratory work	Difficult to assemble PES membrane needs to be humid High solvent use Requires manual skills Qualitative Specialized personnel for laboratory work	Difficult to assemble Limited to polar range Sorbent loss Requires manual skills Qualitative Specialized personnel for laboratory work	Instantaneous Limited to hydrophobic range Potential environmental contamination Expensive Destructive sample	Instantaneous Labour-intensive process Sample loss Expensive Specialized personnel for laboratory work
	Suggestions	Casing needs to be adapted to thicker disks Deployment should be made easier	Deployment should be made easier	Deployment should be made easier	Expand polarity range Investigate matrix effects	Shorten and simplify the procedure

POCIS: Polar Organic Chemical Integrative Sampler; SBSE: Stir-Bar Sorptive Extraction; SPE: Solid-Phase Extraction; HLB: Hydrophilic–Lipophilic Balance (HLB); SDB: Styrene–Divenylbenzene; PDMS: Polydimethyl Siloxane, PES: Polyethersulfone, PTFE: Polytetrafluoroethylene. The background colors have been used to differentiate different criteria used for the assessment of the sampling and extraction techniques. N.A. stands for Not Applicable.

## Data Availability

The original contributions presented in this study are included in the article/Supplementary Materials. Further inquiries can be directed to the corresponding author.

## Supplementary Materials

The following supporting information can be downloaded at: https://www.mdpi.com/article/10.3390/molecules31030529/s1: Table S1: Sampling coordinates; Table S2: List of screened pesticides and their category, retention time, quantifier and up to 3 qualifier ions; Table S3: Graphical summary of sample preparation, processing and analysis; Table S4: Details of GC-QToF-MS acquisition methods by desorption and injection; Table S5: List of compounds detected at each site, with corresponding Log K_ow_ and frequency values (where applicable).

## Abbreviations

The following abbreviations are used in this manuscript:

POCIS	Polar Organic Chemical Integrative Sampler
SBSE	Stir-Bar Sorptive Extraction
SPE	Solid-Phase Extraction
AUD	Australian Dollars
NMI	National Monitoring Institute
EDC	Endocrine-Disrupting Chemicals
RMIT	Royal Melbourne Institute of Technology
AQUEST	Aquatic Environmental Stress Research Group
GC	Gas Chromatography
HLB	Hydrophilic–Lipophilic Balance
PES	Polyether Sulfone
SDB-XC	Polystyrene–DivinylBenzene
PDMS	Polydimethyl Siloxane
HDPE	High-Density Polyethylene
GC-QToF-HRMS	Gas Chromatography Quadruple Time-of-Flight High-Resolution Mass Spectrometry
MPS	Multi-Purpose Sampler
TDU2	Thermal Desorption Unit
